# Protection from successive Omicron variants with SARS-CoV-2 vaccine and monoclonal antibodies in kidney transplant recipients

**DOI:** 10.3389/fmicb.2023.1147455

**Published:** 2023-03-29

**Authors:** Valérie Moal, Margaux Valade, Céline Boschi, Thomas Robert, Nicolas Orain, Audrey Bancod, Sophie Edouard, Philippe Colson, Bernard La Scola

**Affiliations:** ^1^Aix Marseille Université, Institut de Recherche pour le Développement, Microbes Evolution Phylogeny and Infections (MEPHI), Assistance Publique Hôpitaux de Marseille, Marseille, France; ^2^Aix Marseille Université, Assistance Publique Hôpitaux de Marseille, Hôpital Conception, Centre de Néphrologie et Transplantation Rénale, Marseille, France; ^3^Institut Hospitalo-Universitaire (IHU) Méditerranée Infection, Assistance Publique Hôpitaux de Marseille, Marseille, France

**Keywords:** SARS-CoV-2, COVID-19, kidney transplantation, immunocompromized, vaccine, tixagevimab, cilgavimab, neutralization

## Abstract

**Introduction:**

Kidney transplant recipients (KTRs) are at high risk of severe COVID-19, even when they are fully vaccinated. Additional booster vaccinations or passive immunization with prophylactic monoclonal antibodies are recommended to increase their protection against severe COVID-19.

**Methods:**

Here, we describe the neutralization of SARS-CoV-2 Delta, Omicron BA.1, BA.2, BA.4, and BA.5 variants, firstly by 39 serum samples from vaccinated KTRs exhibiting anti-spike antibody concentrations ≥264 binding antibody units (BAU)/mL and, secondly, by tixagevimab/cilgavimab.

**Results:**

No neutralization was observed for 18% of the KTRs, while serum from only 46% of patients could neutralize the five variants. Cross-neutralization of the Delta and Omicron variants occurred for 65–87% of sera samples. The anti-spike antibody concentration correlated with neutralization activity for all the variants. The neutralization titers against the Delta variant were higher in vaccinated KTRs who had previously presented with COVID-19, compared to those KTRs who had only been vaccinated. Breakthrough infections occurred in 39% of the KTRs after the study. Tixagevimab/cilgavimab poorly neutralizes Omicron variants, particularly BA.5, and does not neutralize BQ.1, which is currently the most prevalent strain.

**Discussion:**

As a result, sera from seropositive vaccinated KTRs had poor neutralization of the successive Omicron variants. Several Omicron variants are able to escape tixagevimab/cilgavimab.

## 1. Introduction

Kidney transplant recipients (KTRs) have been proven to be highly vulnerable to COVID-19, exhibiting a mortality rate of approximately 20%, considerably higher than that of the general population (Caillard et al., 2020, 2021b; Jager et al., 2020; Williamson et al., 2020). These individuals were, therefore, prioritized for vaccination with either of the two approved SARS-CoV-2 mRNA vaccines against the causative virus, severe acute respiratory syndrome coronavirus 2 (SARS-CoV-2), by the French health authorities (HAS, Haute Autorité de Santé, 2020). Nonetheless, severe cases of COVID-19 in vaccinated KTRs and reduced response rates were reported following a standard two-dose vaccine regimen (Benotmane et al., 2021; Boyarsky et al., 2021; Rincon-Arevalo et al., 2021; Sattler et al., 2021; Caillard et al., 2021a). To increase the efficacy of vaccination against SARS-CoV-2 with a view to preventing severe COVID-19 among KTRs, administration of a third vaccine dose was recommended (Levin et al., 2021; Mizrahi et al., 2021; Shrotri et al., 2021). Other approaches involved using commercially available serological assays for KTRs to assess humoral responses after vaccination and to guide routine clinical practice decisions regarding additional booster vaccination(s), and passive immunization with prophylactic monoclonal antibodies (mAb) in the event of an insufficient immune response after a complete vaccination schedule. Thus, Feng et al. showed that a vaccine efficacy of 80% against symptomatic infection with the majority Alpha (B.1.1.7) variant of SARS-CoV-2 was achieved with 264 binding antibody (Ab) units (BAU)/mL (Feng et al., 2021). This protection threshold of 264 BAU/mL was established in the context of infections with the Alpha and Beta variants but was considered a minimum level against the Delta variant. The French health authorities recommended the use of anti-spike Ab concentrations as a correlate of protection against SARS-CoV-2 infection (COSV, Conseil d’Orientation de la Stratégie Vaccinale, 2021; SFNDT SFT, Société Francophone de Néphrologie, Dialyse et Transplantation, Société Francophone de Transplantation, 2021; COSV, 2022). Currently, a patient who presents an Ab titer below 264 BAU/mL in France is considered as a non-responder or a weak responder to vaccination (SFNDT SFT, 2022a,b) and may receive a combination of tixagevimab and cilgavimab human mAb, for SARS-CoV-2 preexposure prophylaxis as alternative humoral protection against an inefficient vaccine response. This is similar to the situation in the US and various other European countries (COSV, Conseil d’Orientation de la Stratégie Vaccinale, 2021; Levin et al., 2022). However, tixagevimab/cilgavimab has been shown to inhibit SARS-CoV-2 variants differently (Boschi et al., 2022; Bruel et al., 2022; Touret et al., 2022). The most recent variants of concern, including Delta and then Omicron, have been associated with considerable immune escape and have increasingly led to breakthrough infections, raising questions about the efficacy of booster doses or mAb (Miyamoto et al., 2022; Servellita et al., 2022). In this study, we first aimed to determine whether serum samples from KTRs exhibiting anti-spike Ab concentrations above 264 BAU/mL were effective in neutralizing recent Omicron variants. In the second part, we tested the neutralizing potency of mAb tixagevimab and cilgavimab, either alone or in combination, against multiple recent Omicron variants.

## 2. Materials and methods

### 2.1. Study population and design

KTRs were included if they were followed at the Centre de Néphrologie et Transplantation Rénale within the Marseille University Hospitals in France, and had received at least one dose of vaccine against SARS-CoV-2 and no preventive treatment with a combination of tixagevimab/cilgavimab before sampling for serology. Their levels of anti-SARS-CoV-2 spike Ab assessed in the microbiology laboratory of Marseille University hospitals also had to be above 264 BAU/mL, and the results of neutralization assays had to be available.

The patients’ demographic characteristics, medical history, any prior COVID-19 infection, and information on the modalities of vaccination against COVID-19 were collected in the context of routine clinical patient care from medical records. SARS-CoV-2 infections were documented by PCR or positive serology against the viral nucleocapsid. Data were anonymized, approved according to the General Data Protection Regulation, and registered with the Health Data Portal and Data Protection Commission of the Assistance Publique-Hôpitaux de Marseille under reference PADS22-136. Patients were provided with written information about this study. This study was approved by the Ethics Committee of the IHU Méditerranée Infection under number 2022–003.

### 2.2. Assessment of humoral immune responses directed against SARS-CoV-2 by chemiluminescence and Western blot

As part of routine treatment, in line with the recommendations of the transplant societies and the French health authorities (COSV, Conseil d’Orientation de la Stratégie Vaccinale, 2021; SFNDT SFT, Société Francophone de Néphrologie, Dialyse et Transplantation, Société Francophone de Transplantation, 2021), we monitored serological responses in KTRs. All serological tests were assessed in the microbiology laboratory of Marseille University hospitals. When the KTRs had more than one serological test, we considered the most recent serology result. Sera were tested by automated serology using the Liaison SARS-CoV-2 TrimericS IgG assay, a chemiluminescence immunoassay (Diasorin Inc., Saluggia, Italy), to determine the concentrations of IgG to the Trimeric spike glycoprotein of SARS-CoV-2 in BAU/mL. Concentrations ≥33.8 BAU/mL were considered positive (detection range: 5–2,080 BAU/mL). For sera with IgG concentrations >2,080 BAU/mL, dilutions were carried out, and the results were multiplied by the dilution factor. According to the manufacturer, the results of the Liaison assay strongly correlate with those of the microneutralization test (positive agreement: 100%, negative agreement: 96.9%).

The JessTM Simple Western automated nanoimmunoassay system (ProteinSimple, San Jose, CA, USA, a Bio-Techne Brand), involving capillary-based size separation of proteins, was used to evaluate the absolute serological response to five viral antigens, four from the spike and one from the nucleocapsid (Davoust et al., 2022). The presence of anti-spike IgG indicated vaccination and/or prior SARS-CoV-2 infection. IgG against the nucleocapsid, which is not used as a target in the vaccine, indicated prior COVID-19 infection.

### 2.3. Neutralization assays

Neutralization of SARS-CoV-2 variants of interest was assessed using a live-virus assay. Vero E6 cells (ATCC-CRL-1586) and Vero E6/TMPRSS2 cells (NIBSC 100978) were cultured under previously described conditions, except that 1 mg/mL geneticin was added to the medium for the former cells (Boschi et al., 2022). Viruses were isolated from patients’ nasopharyngeal swabs taken during the pandemic, and genotyping was performed from the supernatants by whole-genome next-generation sequencing (NGS), as previously described (Colson et al., 2021; Boschi et al., 2022).

Sera and mAb were diluted in a serial dilution (from 1:5 to 1:640 for sera and from 10,000 μg/mL to 0.0512 μg/mL for tixagevimab, cilgavimab and the combination of both), as previously described (Boschi et al., 2022). Each dilution was mixed volume-to-volume with a viral inoculum quantified at a cycle threshold value (Ct) of 25 by real-time reverse-transcription (RT)-PCR (qPCR) and by TCID_50_ determination (4.29 ± 4.13 log_10_ virus/mL for all strains). The neutralization titer was determined 5 days post-viral infection by observing the cytopathic effect with an inverted optical microscope. The highest serum dilution that resulted in more than 50% inhibition of the cytopathic effect was defined as the sample’s neutralization titer. Each serum was tested once against the Delta variant (Pangolin lineage AY.71), the Omicron BA.1 variant (B.1.1.529.1), the Omicron BA.2 variant (B.1.1.529.2), the Omicron BA.4 (B.1.1.529.4) and the Omicron BA.5 variant (B.1.1.529.5). Accession numbers of the strains are available in Table 1. Serum neutralizing activity was detectable if the neutralization titer was ≥ 5. Tixagevimab, cilgavimab, and the combination of both were tested four times against the Omicron BA.2, BA.4, BA.5 variants and BQ.1, which is derived from BA.5. The combination of tixagevimab and cilgavimab was considered as a control on the Omicron BA.1 variant, as previously described (Boschi et al., 2022). Each condition was tested in quadruplicate in 96-well plates.

**Table 1:** Accession numbers of isolates of SARS-CoV-2.

Pangolin lineage	Nextstrain (WHO) clade	Sequences names
AY.71	21J (Delta)	IHUMI-3630_EPI_ISL_8033350|2021-06-07
B.1.1529	21K (Omicron)	IHUMI-5227_EPI_ISL_8033859|2021-12-01
B.1.1529.2	21l (Omicron BA.2)	ON649998
B.1.1529.4	22A (Omicron BA.4)	PRJEB55106
B.1.1529.5	22B (Omicron BA.5)	ON898599.1
B.1.1.529.5.3.1.1.1.1.1	22E (Omicron BQ.1)	OQ155176

### 2.4. Statistics

Continuous and categorical variables were expressed as the medians (interquartile range (IQR)) and n (%), respectively. We used Student’s t test, *χ*^2^ test and Tukey’s multiple comparisons test as appropriate to compare differences between groups. All tests were two-tailed. Statistical significance was assumed at *p* < 0.05. The relationship between the concentration of anti-spike Ab and the neutralization titer was analyzed by simple linear regression. Correlation was performed by Pearson’s analysis. All statistical analyses were performed using GraphPad PRISM software.

## 3. Results

### 3.1. Patients

**Figure 1:**
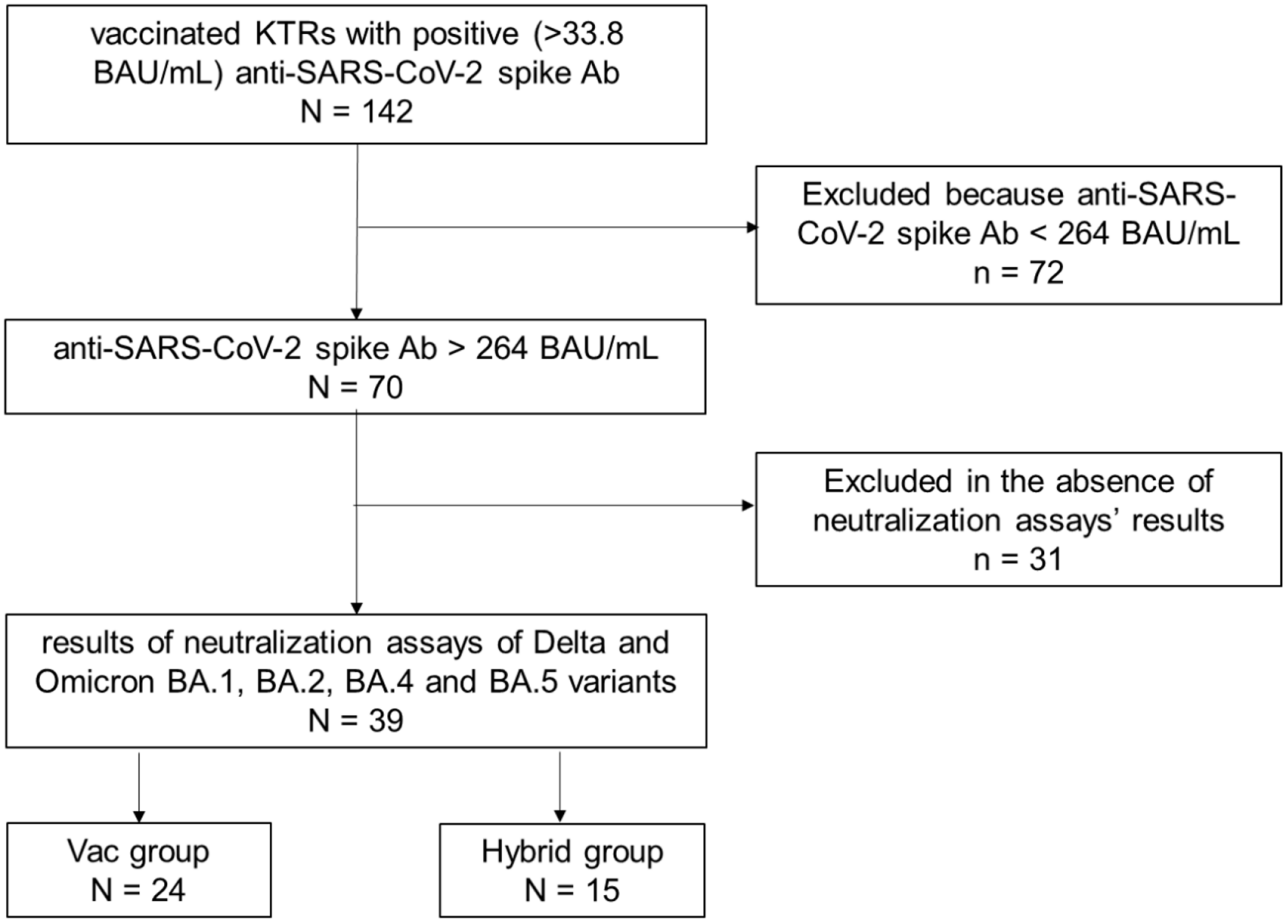
Flow chart. Between August 23, 2021 and April, 262,022, 142 kidney transplant recipients (KTRs) received at least one dose of vaccine against SARS-CoV-2 before sampling for serology and had positive (> 33.8 binding antibody (Ab) units (BAU)/mL) anti-SARS-CoV-2 spike Ab in our laboratory. Of the 142 KTRs, 72 had anti-spike Ab concentrations <264 BAU/mL, and 70 had concentrations >264 BAU/mL. Neutralization assays results were available for 39 of these 70 KTRs and were included in the study. The Hybrid group is formed of 15 KTRs immunized against SARS-CoV-2 by vaccination and prior COVID-19, and the Vac group is formed of 24 KTRs immunized only by vaccination.

Between August 23, 2021 and April 26, 2022, 142 KTRs received at least one dose of vaccine against SARS-CoV-2 before sampling for serology and revealed a positive (> 33.8 BAU/mL) anti-SARS-CoV-2 spike Ab in our laboratory. Of the 142 KTRs, 72 had anti-spike Ab concentrations < 264 BAU/mL, and 70 had concentrations > 264 BAU/mL. Neutralization assay results were available for 39 of these 70 KTRs. We therefore included 39 KTRs in the study (Figure 1). Their main characteristics at the time of serology are presented in Table 2. At the time of serology, the median age of the 14 women and 25 men was 58 years (IQR, 40–67), and the median duration since the last kidney transplantation was 6.9 years (0.9–14.7). All but two KTRs received calcineurin inhibitors and 35 of the 39 received corticosteroids. No KTR had received preventive treatment with a combination of tixagevimab and cilgavimab before sampling for serology.

**Table 2:** Characteristics of the 39 kidney transplant recipients at the time of serology.

Number	39
Male/female	25/14
Median age in years (IQR)	58 (40–67)
Median time from kidney transplantation in years (IQR)	6.9 (0.9–14.7)
Number of kidney transplantations *n* = 1 *n* = 2	33 (one simultaneous kidney and pancreas transplantation) 6
Acute rejection of the current transplantation	3
Median time from acute rejection in days (range)	94 (32–4,611)
Median concentration of anti-spike Ab in BAU/mL (IQR)	1,630 (968–4,590)
Number of prior COVID-19 (%)	15/39 (38%)
Median time from COVID-19 in days^a^ (IQR)	237 (88–373)
Median vaccine dose number (IQR)	3 (3–4)
Median estimated GFR in mL/min/1.73 m^2^ (IQR)	46 (32–57)

a Two kidney transplant recipients without prior PCR-proven COVID-19 were shown to have positive Western blot serology against the nucleocapsid protein, with no possibility to date the infection. Median time from COVID-19 was calculated for the 13 others. Interquartile (IQR).

The 39 participants included KTRs who had received at least one dose of vaccine against SARS-CoV-2 and their median vaccine dose number before sampling for serology was 3 (IQR, 3–4). Messenger RNA vaccines were mostly BNT162b2 (Pfizer-BioNTech) for 108 doses and mRNA-1,273 (Moderna) for 11 doses. Only two doses of the AstraZeneca vaccine were given. The 39 KTRs were divided in two groups according to whether or not they had presented COVID-19 before sampling for serology (Table 3). Thirteen KTRs had presented COVID-19 as evidenced by a PCR test before sampling: four between May and November 2020, seven between January and November 2021, and two between January and February 2022. For 3/13 patients, SARS-CoV-2 was genotyped (B.1.177, B.1.160, and Delta variants). In the 10 other cases, COVID-19 was PCR-diagnosed in independent laboratories, and the virus genotype was not available. Sera from the 39 KTRs were tested using an automated Western blot assay to detect undiagnosed COVID-19. All patients reacted against the spike viral protein. Two KTRs without prior PCR-proven COVID-19 were shown to have positive serology against the nucleocapsid protein, suggesting prior paucisymptomatic infection with no possibility to date the infection. Therefore, the group of KTRs with prior COVID-19 before entering the study consisted of 15 patients (38.5%) and formed the “hybrid” group, so-called due to their hybrid immunization against SARS-CoV-2. Twenty-four KTRs (61.5%) were immunized against SARS-CoV-2 due to vaccination only and formed the “Vac” group.

**Table 3:** Characteristics of the Hybrid and Vac groups at the time of serology.

	Hybrid group	Vac group
*N* (%)	**15**/39 (38.5%)	**24**/39 (61.5%)
Male/female	11/4	14/10
Median age in years (IQR)	53 (50–66)	60 (38–67)
Median time from kidney transplantation in years (IQR)	7 (2–11)	9 (0.7–15)
Number of kidney transplantations *n* = 1 *n* = 2	13 2	20 4
Median time from COVID-19 in days^a^ (IQR)	237 (88–373)	-
Transplanted before COVID-19^b^	13 or 14/15 (86% or 93%)	-
Vaccinated before COVID-19^b^ (%)	7/13 (54%)	-
Median vaccine dose number before COVID-19^a^ (IQR)	2 (0–2)	-
Median vaccine dose number before serology (IQR)	3 (2–4)	3 (3–4)
Median time from the last known immunizing event before serology in days^c^ (IQR)	84 (60–95)	99 (67–134)
Median estimated GFR in mL/min/1.73 m^2^ (IQR)	38 (31–60)	48 (31–57)
Median concentration of anti-spike Ab in BAU/mL (IQR)	1,830 (1,231–8,425)	1,385 (984–1,997)
N neutralizing ≥ one variant (%)	14/15 (93%)	18/24 (75%)

a Two kidney transplant recipients (KTRs) without prior PCR-proven COVID-19 were shown to have positive Western blot serology against the nucleocapsid protein, with no possibility to date the infection. Median time from COVID-19 was calculated for the 13 others.

b One of these 2 KTRs diagnosed with prior COVID-19 using Western blot has been transplanted before the pandemic. For the second one, it is impossible to know the temporality between COVID-19 and kidney transplantation. Thus, the numerator is 13 or 14.

c Immunizing events were vaccination or COVID-19. Interquartile (IQR).

### 3.2. Neutralizing activity of the sera against the Delta and Omicron BA.1, BA.2, BA.4, and BA.5 variants

We assessed the neutralizing activity of each serum sample from the 39 KTRs using a live-virus assay against the last successive SARS-CoV-2 variants of interest, the Delta and Omicron BA.1, BA.2, BA.4 and BA.5 variants. Despite anti-spike Ab concentrations > 264 BAU/mL, impaired neutralization against these five variants of SARS-CoV-2 was observed. Indeed, 18/39 (46.1%) KTRs neutralized the five variants, but 7/39 (17.5%) neutralized, at most, four variants, 3/39 (7.7%) three variants, 1/39 (2.6%) two variants, and 3/39 (7.7%) one variant. Finally, no neutralization was observed in 7/39 (17.9%) KTRs, regardless of the variant. We compared gender, age, time from kidney transplantation, number of kidney transplantations, number of acute rejections, concentration of anti-spike Ab, prior COVID-19, time from COVID-19, vaccine dose number, time from first and last vaccine dose, and estimated GFR between the KTRs who neutralized at least one virus (n = 32) and those who neutralized no virus (n = 7) to test whether the host’s characteristics could explain the lack of neutralizing activity. The only difference detected between the two groups was regarding the median concentration of anti-spike Ab, which was significantly higher in the KTRs who neutralized at least one virus (1,730 BAU/mL, IQR, 1,247–9,862) than in the KTRs who neutralized no virus (555 BAU/mL, 505–778; *p* = 0.02). Fourteen of the 32 KTRs who neutralized at least one virus presented prior COVID-19 *vs* only one KTR among the seven who neutralized no virus (*p* = 0.15). This was a 31-year-old man who was transplanted 348 days before sampling for serology. He had been treated with tacrolimus, azathioprine, prednisone and eculizumab, and had been vaccinated with four doses of the BNT162b2 vaccine. COVID-19 was retrospectively diagnosed by Western blotting. His anti-spike Ab concentration was 551 BAU/mL at the time of the study. The six other KTRs who neutralized no virus received either three or four doses of vaccine.

**Figure 2:**
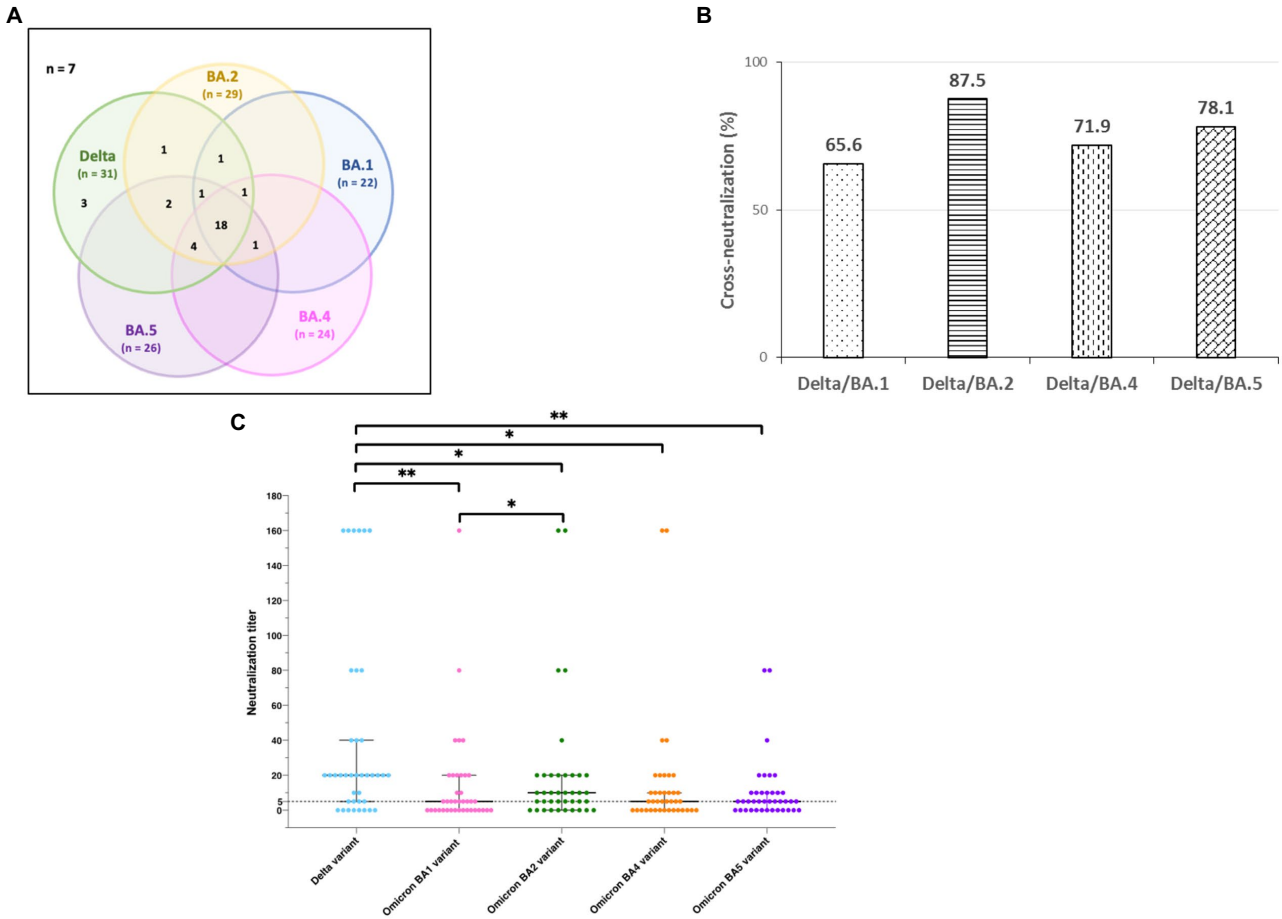
Neutralization of SARS-CoV-2 variants by sera from 39 kidney transplant recipients. **(A)** Profiles of SARS-CoV-2 variant neutralization and cross-neutralization by sera from 39 kidney transplant recipients. The cross-neutralizations are shown in a Venn diagram. Numbers in parentheses are the total numbers of sera that neutralized the variant. Seven sera had no neutralizing activity against the five SARS-CoV-2 variants, whereas 32 neutralized at least one variant. Eighteen neutralized all the variants. **(B)** Frequencies of cross-neutralization of Delta and different Omicron variants for the 32 sera that neutralized at least one variant. **(C)** Neutralization titers in the sera from 39 kidney transplant recipients according to the SARS-CoV-2 variant. The black bar represents the median value, and error bars represent the interquartile ranges of values. The black dashed line represents the cutoff for a detectable neutralization titer of 5. The neutralization titers against the five variants were compared using one-way ANOVA followed by Tukey’s multiple comparison test. *p < 0.05, **p < 0.002.

Sera from KTRs showed better neutralization against the Delta than the Omicron variants. The most frequently neutralized SARS-CoV-2 variant by sera of KTRs was the Delta variant (31/39, 79.5%), followed, in decreasing order, by the Omicron BA.2 (29/39, 74.4%), BA.5 (26/39, 66.7%), BA.4 (24/39, 61.5%) and, finally, BA.1 (22/39, 56.4%) variants. The profiles of neutralization of SARS-CoV-2 variants by sera of the 39 KTRs and of cross-neutralization are illustrated in Figure 2A. Considering the 32 neutralizing sera, cross-neutralization of the five variants was observed in 18 (56%) cases. Cross-neutralization of the Delta and Omicron BA.2 variants was frequent in 28 (88%) cases, and less frequent cross-neutralization was observed between the Delta and Omicron BA.1 variants in 21 (66%) cases. Nevertheless, there was no statistically significant cross-neutralization of Delta and different Omicron variants (Figure 2B, *p* = 0.2). The median neutralization titers were significantly different against the five variants: 20 (IQR, 20–40) for Delta, five (0–20) for BA.1, 10 (2.5–20) for BA.2, five (0–10) for BA.4, and BA.5 (*p* < 0.0001) (Figure 2C). They were significantly higher against the Delta variant than against all Omicron variants, particularly BA.1 and BA.5. Neutralization titers were significantly lower against BA.1 than BA.2.

**Figure 3:**
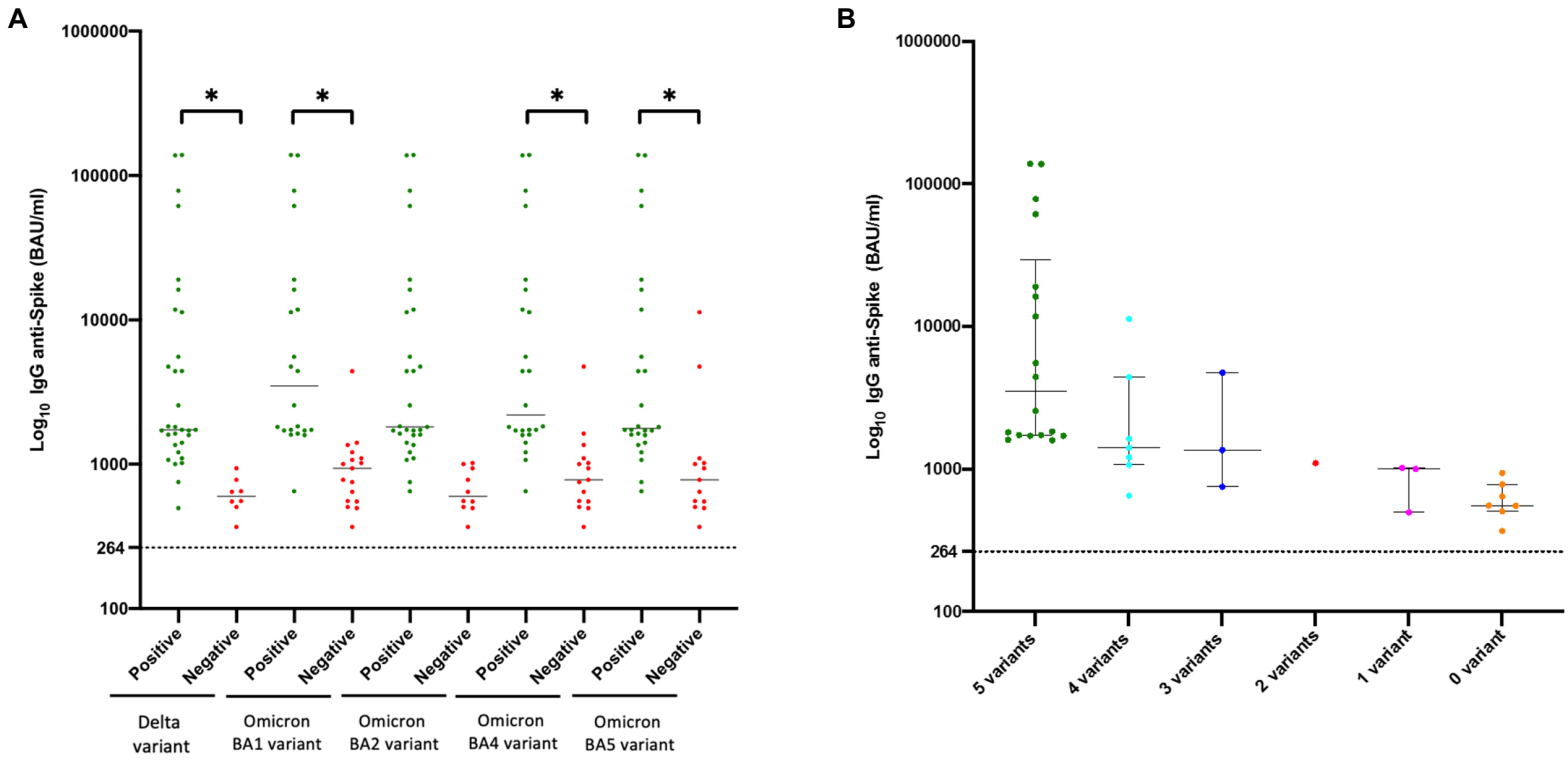
Anti-SARS-CoV-2 humoral immunity in 39 kidney transplant recipients according to anti-spike antibody concentrations. Concentrations of anti-SARS-CoV-2 IgG are expressed in Log10BAU/mL. The black bar represents the median value **(A)**. Concentrations of anti-SARS-CoV-2 IgG were compared between sera with and without neutralizing activity for each virus using one-way ANOVA followed by Tukey’s multiple comparison test; *p < 0.05. Each point represents one serum sample. The point is green if the serum presented neutralizing activity and red if not. **(B)** Concentrations of anti-SARS-CoV-2 IgG were compared between sera neutralizing different numbers of variants.

**Figure 4:**
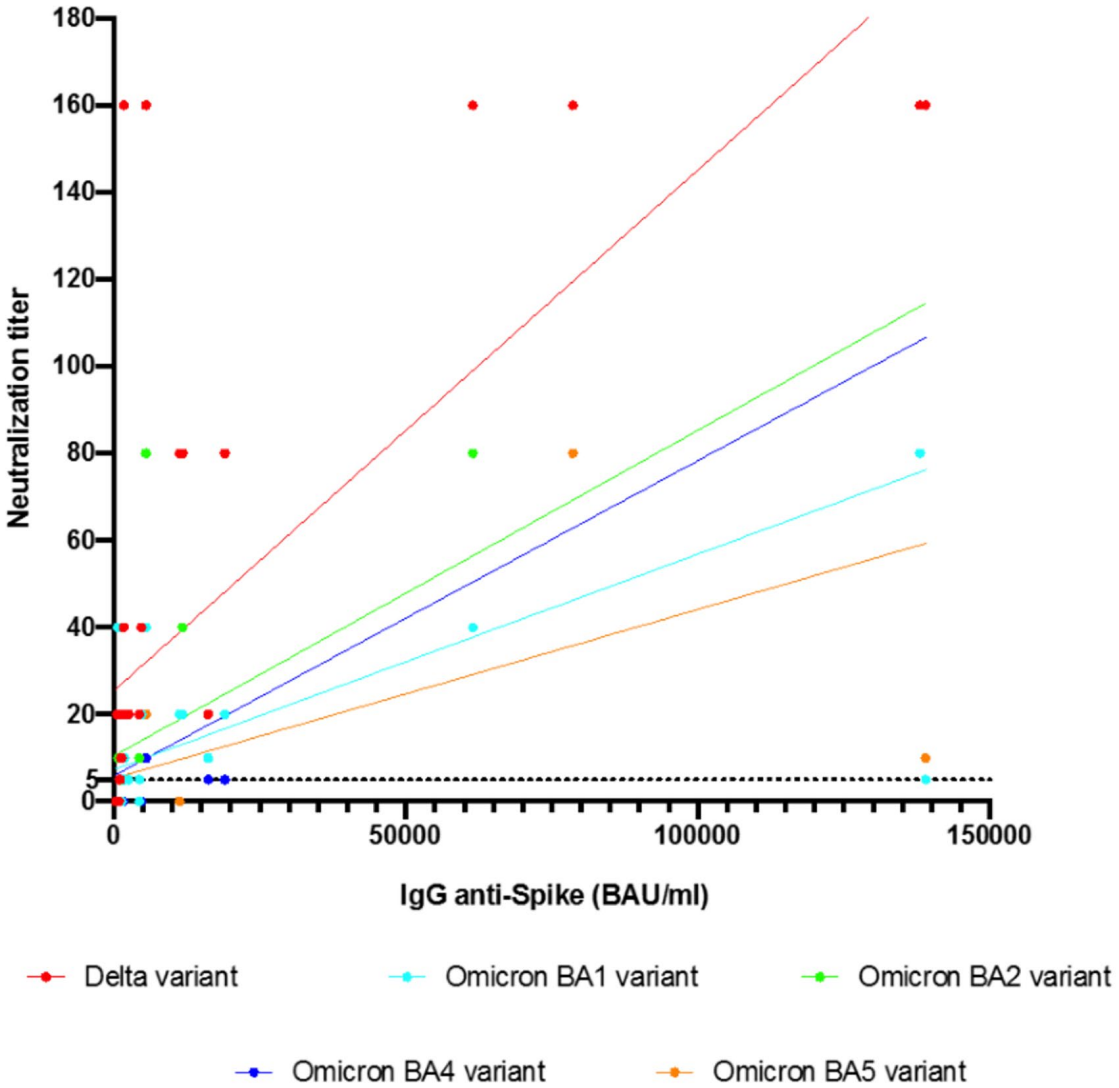
Correlation analyses of anti-spike Ab with neutralization titers of the sera from 39 kidney transplant recipients against the different SARS-CoV-2 variants by simple linear regression. The correlation was performed by Pearson’s analysis. The concentrations of anti-spike Ab showed a strong correlation with the neutralization titer for each variant. The Pearson r was 0.72, 0.57, 0.67, 0.68, and 0.71 for the Delta, Omicron BA.1, Omicron BA.2, Omicron BA.4, and Omicron BA.5 variants, respectively; *p* < 0.0001.

The anti-spike Ab concentration correlated with neutralization activity. The median concentration of anti-spike Ab in the 39 KTRs was 1,630 BAU/mL (IQR, 968–4,590). As mentioned above, it was significantly higher in KTRs who neutralized at least one SARS-CoV-2 variant than in KTRs without SARS-CoV-2 neutralization. This was also true in KTRs who neutralized viruses regardless of the variant compared to KTRs who did not (Figure 3A). The number of different variants neutralized seemed to increase with the anti-spike IgG concentration (Figure 3B). Anti-spike Ab concentrations were strongly correlated with the neutralization titer for each variant (r = 0.72, 0.57, 0.67, 0.68, and 0.71 for Delta, BA.1, BA.2, BA.4, and BA.5 variants, respectively; *p* < 0.0001) (Figure 4).

**Figure 5:**
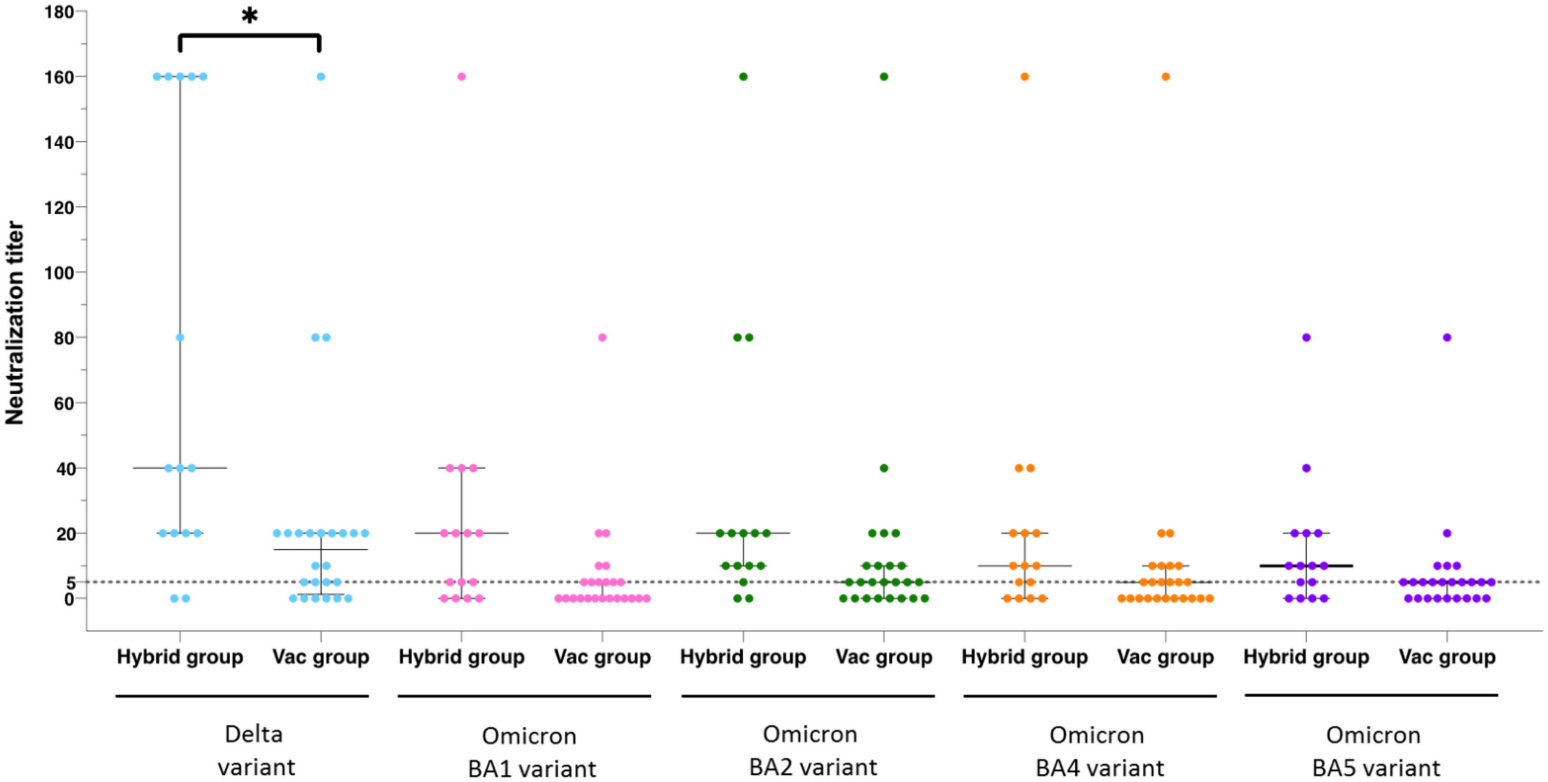
Neutralization titers in the Hybrid group and the Vac group according to the SARS-CoV-2 variants. Neutralization titers were compared for each variant between the Hybrid group, formed of kidney transplant recipients immunized against SARS-CoV-2 by vaccination and prior COVID-19, and the Vac group, formed of patients immunized only by vaccination, using one-way ANOVA followed by Tukey’s multiple comparison test; *p < 0.05.

We assessed the anti-spike Ab concentrations and neutralizing activity in the Hybrid and Vac groups (Table 2). Fourteen (93%) of the 15 KTRs in the Hybrid group neutralized ≥ 1 virus compared to 18/24 (75%) in the Vac group. The median anti-spike Ab concentration was no different between the groups (1,830 (IQR, 1,231–5,550) vs. 1,385 (984–1,997) BAU/mL, respectively; *p* = 0.32). When we compared the median neutralization titers against the five variants between the two groups, they were significantly higher in the Hybrid group than in the Vac group only for the Delta variant (40 (20–160) vs. 15 (1.25–20), respectively; *p* = 0.017) (Figure 5). Interestingly, three cases displayed the highest neutralization titer against the variant they were suspected to have been infected with. Our observations suggest that infection with the Delta or Omicron BA.1 variants could lead to neutralization of these variants, despite anti-spike Ab concentrations being lower than those observed in patients who had been immunized only by vaccination.

### 3.3. Breakthrough infections

**Figure 6:**
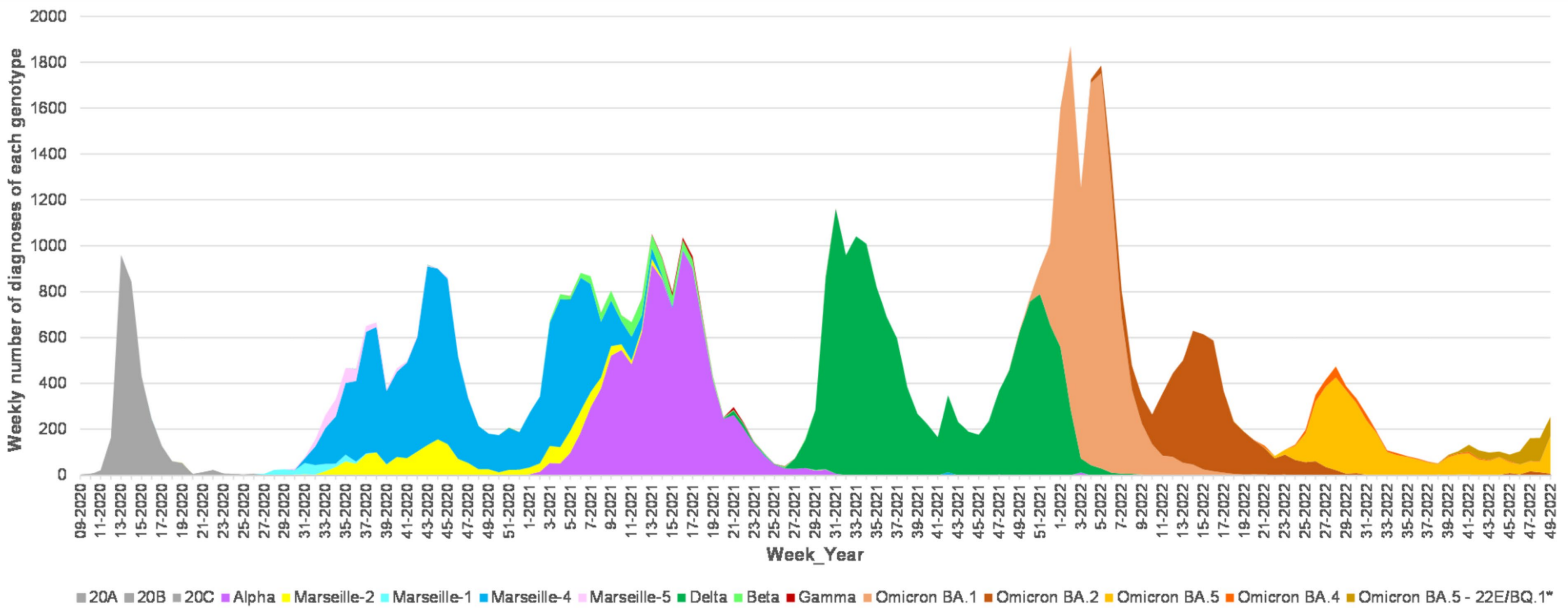
Weekly incidence of each major SARS-CoV-2 mutant and variant based on the genotyping performed at IHU Méditerranée Infection institute. SARS-CoV-2 genotyping was performed as previously described (Colson et al., 2022). Correspondence between genotype labels: Marseille-1: Pangolin lineage (Rambaut et al., 2020) B.1.416; Marseille-2: B.1.177; Marseille-4: B.1.160; and Marseille-5: B.1.367.

**Figure 7:**
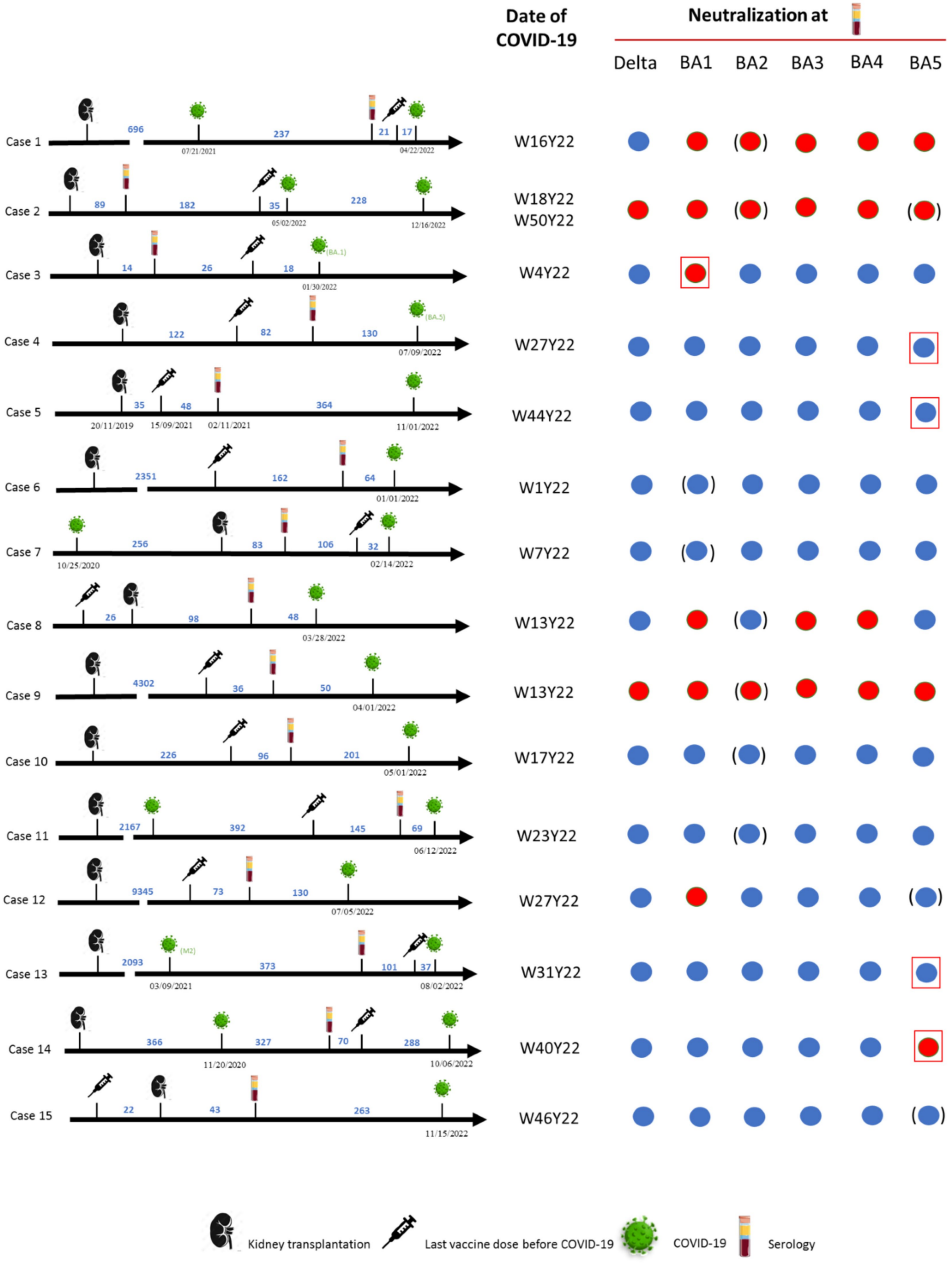
Breakthrough infections after seroneutralization study. Chronograms including events such as kidney transplantation, the last dose of vaccine before COVID-19, serology and COVID 19, are represented for 15 of the 38 patients, all of whom were kidney transplant recipients, at the last follow-up (December 28, 2022). The date of COVID-19 is given in form of Week (W) and Year (Y) numbers. The results of the seroneutralization of the Delta, Omicron BA.1, BA.2, BA.4 and BA.5 variants at the time of the serology are presented: the disk is blue if neutralization was observed, and red if not. If the virus responsible for COVID-19 was genotyped at our laboratory, the concerned variant in question is framed by a red square. In other cases, the supposed variant is circled by blue brackets, according to the weekly incidence of each major SARS-CoV-2 mutant and variant, based on the genotyping performed at our laboratory.

SARS-CoV-2 neutralization was analyzed in sera sampled between September 2021 and April 2022. At a median of 321 days (IQR, 288–441) after the study, 15/38 (39%) KTRs had developed COVID-19. All infections occurred in 2022 when the Omicron variants were predominant in France and on the genotyping performed in the microbiology laboratory of Marseille University hospitals (Figure 6). Case #2 presented two episodes of COVID-19 suspected to be linked first to Omicron variant BA.2 then to BA.5 (Figures 6, 7). The viral genotype was determined in our laboratory for only the cases #3, #4 and #5. Case #3 was infected in January by Omicron BA.1, 2 months after kidney transplantation and 18 days after a fourth vaccine dose, with a paucisymptomatic form and PCR positivity for 2 months post-sotrovimab treatment. The anti-spike Ab concentration of the serum sampled 44 days before COVID-19 was 1,070 BAU/mL and all the variants were neutralized except Omicron BA.1 (Figure 7). Case #4 was infected in July by Omicron BA.5 11 months after kidney transplantation and 7 months after a third vaccine dose. This patient died of severe pneumopathy. The serum sampled 130 days before COVID-19 neutralized all the variants, and the neutralization titer against BA.5 was 5. The last anti-spike Ab concentration, 2.5 months before COVID-19 was 1,070 BAU/mL. Case #5 was infected in November by Omicron 22B/BF.7 variant, which is derived from BA.5, 15 months after kidney transplantation and 14 months after a third vaccine dose. This patient required hospitalization. The serum sampled 364 days before COVID-19 neutralized all the variants, and the neutralization titer against BA.5 was 5. The last anti-spike Ab concentration, 6.5 months before COVID-19, was 750 BAU/mL. The genotypes of the viruses infecting the 12 other patients were not available, however given the date of infection and the weekly incidence of each major SARS-CoV-2 mutans and variant based on the genotyping performed at our laboratory, we can assume which genotype was responsible for their infection and observe that cases #1, 2, 3, 9, and 14 did not neutralize the variant they were ultimately assumed to harbor (Figure 7).

### 3.4. Neutralizing activity of the mAbs

**Figure 8:**
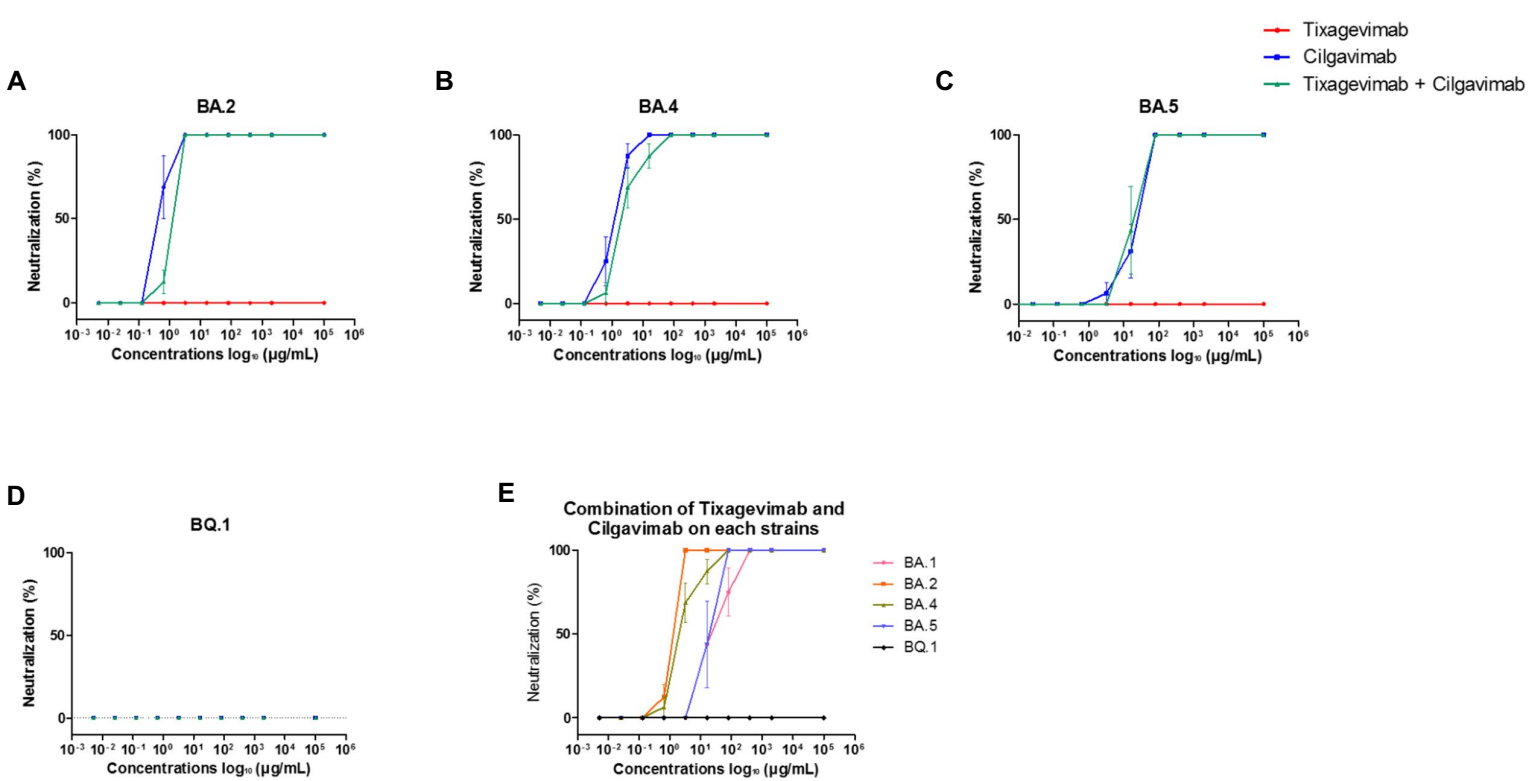
Neutralization curves for Omicron BA.2, BA.4, BA.5, and BQ.1 tested with monoclonal antibodies. **(A)** Omicron BA.2 tested with tixagevimab, cilgavimab, and a combination of both. **(B)** Omicron BA.4 tested with tixagevimab, cilgavimab, and a combination of both **(C)** Omicron BA.5 tested with tixagevimab, cilgavimab, and a combination of both. **(D)** Omicron BQ.1 tested with tixagevimab, cilgavimab, and a combination of both. **(E)** Comparison of each Omicron isolate tested with the tixagevimab/cilgavimab combination; BA.1 was considered as a control. Each experiment was performed four times. Bars represent the standard error.

As the combination of tixagevimab and cilgavimab is currently used for SARS-CoV-2 preexposure prophylaxis to provide alternative humoral protection against an inefficient vaccine response, we studied SARS-CoV-2 variant neutralization by the mAb, alone or in combination. We observed no neutralizing activity of tixagevimab on the Omicron BA.2, BA.4, BA.5, and BQ.1 variants (Figures 8A–D), as we also reported on BA.1 (Boschi et al., 2022). The concentrations required to obtain 50% neutralization with cilgavimab were 1.28 μg/mL, 1.92 μg/mL, and 64 μg/mL for Omicron BA.2, BA.4, and BA.5, respectively (Figures 8A–C). Cilgavimab did not neutralize BQ.1 (Figure 8D). We observed 50% neutralization for the combination of both mAbs, as it neutralized both Omicron BA.2 and BA.4 at 3.2 μg/mL. In contrast, 50% neutralization for Omicron BA.5 was observed with mAb combination at 48 μg/mL (Figure 8E), suggesting no synergistic effect on Omicron BA.5. Finally, the combination of tixagevimab and cilgavimab did not neutralize BQ.1 (Figure 8E).

## 4. Discussion

Poor humoral response to vaccination in KTRs is reported but few data exist on the neutralizing capacity of SARS-CoV-2 variants by sera from KTRs. Previous studies involved the Delta variant before and after a third or a fourth vaccine dose (Kamar et al., 2021; Benning et al., 2022; Benotmane et al., 2022; Charmetant et al., 2022; Kumar et al., 2022a,b). In the present study, we concentrated on a special population of KTRs known as “responders to vaccination” and for 38%, as presenting hybrid immunity due to a combination of prior infection and vaccination. We assessed neutralization against Delta but also against contemporary Omicron variants of SARS-CoV-2. Two studies reported results for neutralization against Omicron variants when these variants were beginning to emerge (Benning et al., 2022; Kumar et al., 2022b). Since then, three major Omicron variants succeeded BA.1, and the question of their neutralization is, to our knowledge, unanswered in KTRs. Our study is the first to describe and compare neutralization of Delta, Omicron BA.1, BA.2, BA.4 and BA.5 variants by KTR sera.

We showed that the KTR neutralizing capacity against the latest SARS-CoV-2 variants of concern is only partial, despite immunization by the vaccine and/or a prior infection. Although the anti-spike Ab concentration was correlated with neutralization activity for all the variants, no neutralization was observed in nearly one-fifth of patients, and neutralization of the five variants was observed in less than half KTRs with anti-spike Ab >264 BAU/mL. In our study, no threshold of BAU/mL could be identified to predict neutralization. The threshold of 264 BAU/mL firstly considered a minimum protection threshold is now used to define non-responder or weak responder KTRs to vaccination (COSV, Conseil d’Orientation de la Stratégie Vaccinale, 2021; SFNDT SFT, Société Francophone de Néphrologie, Dialyse et Transplantation, Société Francophone de Transplantation, 2021; COSV, 2022; SFNDT SFT, 2022b).

KTRs showed better neutralizing activity of the Delta than the Omicron variants in terms of frequency and intensity, and cross-neutralization of the Delta and Omicron variants ranged between 65% for BA.1 and 87% for BA.2. As in Kumar’s study, we found no demographic host variables which were significantly associated with variant neutralization (Kumar et al., 2022b). Taken as a whole, our data indicate a preponderant role of the variant in terms of inducing a neutralizing humoral response.

In healthy subjects, breakthrough infections before or after vaccination have been reported to induce elevated immune responses compared to vaccination and cross-neutralization potency against SARS-CoV-2 variants (Bekliz et al., 2022; Miyamoto et al., 2022). In our study, the neutralization titers against the Delta variant were significantly higher in the KTRs who presented hybrid immunization by a combination of vaccination and prior infection than in the KTRs who were only vaccinated. In vaccinated KTRs, a history of infection with the Delta or Omicron BA.1 variant may have led to a better neutralization capacity of Ab elicited through infection against homologous variants than in the context of isolated vaccination. Miyamoto et al. showed that neutralizing activity against the Delta variant was particularly pronounced in healthy subjects who had a vaccine breakthrough infection with the Delta variant, as we observed in our study (Miyamoto et al., 2022). They also reported neutralizing activity against the Omicron variant, in contrast to sera from individuals who had no breakthrough infections, with large inter-individual variations in the degree of neutralizing activity. They suspected that a Delta breakthrough infection was beneficial for the induction of cross-neutralizing Ab to the Omicron variant. We observed cross-neutralization of BA.2 but not of BA.1 in our study. Although Omicron infection has been reported to boost existing immunity conferred by vaccination against other variants in a mouse model, suggesting that “hybrid immunity” is effective against both Omicron and other variants (Suryawanshi et al., 2022), we did not observe neutralization of the Delta variant in case of probable infection by BA.1.

Our study has limitations. The number of patients was relatively small, and we did not test neutralization by KTRs sera of the Omicron BQ.1 variant, which is currently the majority variant. Identification of a viral variant in many KTRs with COVID-19 was absent. Finally, we lack data analyzing cellular immunity. Other immune effectors, in particular T lymphocytes, could contribute toward protection in patients with limited vaccine-induced humoral immunity (KemLin, 2022).

One recent analysis estimated that the risk of reinfection with the Omicron variant was significantly higher than that with the Delta variant in the general population (Miyamoto et al., 2022). In our study, six of the 39 (15%) patients were reinfected, and vaccine breakthrough COVID-19 occurred in 39% of the KTRs during their post-study follow-up in 2022. This may be explained by absent or insufficient neutralizing responses against the Omicron variants, the threshold of neutralizing antibodies correlated to clinical protection being undetermined, and probably not enough for reaching this aim. The prognostic performance and the role of the sera SARS-CoV-2 neutralization test to evaluate the immune response of KTRs after active or passive immunization could be clarified in a prospective study. One patient out of fifteen (7%) who had vaccine breakthrough Omicron BA.5 COVID-19, died. Infection with the Omicron variants is considered to be less severe than infection with the Delta variant. This is not the case for immunocompromised patients, who always present severe disease and require intensive care even when they are vaccinated. In a French multicenter prospective cohort study of critically ill patients admitted for severe COVID-19 between December 2021 and May 2022, those infected with Omicron variant were immunocompromised patients in 43% of cases, half of whom had received an organ transplant. This figure was three times higher than Delta-infected patients (de Prost et al., 2022). Their day-28 mortality rate was 47%, significantly higher than that of non-immunocompromised patients (26%). Immunocompromised patients had been more frequently vaccinated than their non-immunocompromised counterparts (85.9% vs. 40.5%, respectively). Half of vaccinated immunocompromised patients had received three doses of vaccine. However, the proportion of patients with a detectable anti-spike Ab upon admission did not differ between the two groups. The concentration of anti-spike Ab in vaccinated vs. non-vaccinated immunocompromised patients was also not significantly different, whereas it was significantly different between vaccinated and non-vaccinated immunocompetent individuals. Currently, vaccination does not provide universal protection against SARS-CoV-2 variants. As new SARS-CoV-2 variants repeatedly emerge with no clear seasonality, replacing existing variants over a course of just a few weeks, it seems difficult to succeed in achieving such protection. It remains unknown whether additional booster vaccine doses or updated bivalent vaccine booster doses that target recently circulating Omicron sublineages, would enhance the humoral response in immunocompromised patients. In the multistate VISION Network in the US, the protection offered by vaccination during the period when Omicron was predominant was judged moderate even after a three-dose monovalent primary series or booster dose (Britton et al., 2022).

The use of prophylactic mAb therapy falls within the additional protective recommendations for immunocompromised patients, including KTRs. This is why, in a separate experiment, we also tested the neutralizing capacity of cilgavimab alone or with tixagevimab against the Omicron BA.2, BA.4, BA.5 and BA.5-derived BQ.1 variants. No neutralization by tixagevimab was observed, whereas cilgavimab alone or with tixagevimab neutralized the first three variants. However, the efficacy of the tixagevimab/cilgavimab combination against BA.5 was decreased by approximately 15 times compared to that against BA.2 and BA.4. Cilgavimab alone or in combination with tixagevimab did not neutralize BQ.1. We and others reported *in vitro* partial neutralizing activity of the same combination against BA.1 (Boschi et al., 2022; Bruel et al., 2022; Benotmane et al., 2022; Takashita et al., 2022). *In vitro* results have nevertheless been challenged in retrospective studies that showed that KTRs who received 300 mg tixagevimab /cilgavimab as prophylaxis had similar outcomes to patients with vaccine-induced immunization but had significantly fewer infections (both severe and non-severe) compared to KTRs considered as unprotected during the period when BA.1 predominated (Al Jurdi et al., 2022; Bertrand et al., 2022). Loss of *in vitro* antiviral efficacy against BQ.1 of tixagevimab/cilgavimab has been confirmed by other studies and communicated by health agencies (EMA, European medical agency, 2022; Imai et al. 2022). Although it is not yet known to what extent the reduced neutralizing activity translates into reduced benefits for patients, these results point to the need to target COVID-19 prevention measures at the high-risk of critical illness and death among the immunocompromised population.

## 5. Conclusion

In our study, neutralizing activity against the most recent SARS-CoV-2 variants, including Omicron BA.1, BA.2, BA.4 and BA.5, was poorly detected in vaccinated KTRs who seroconverted. Hybrid immunization by combined vaccination and prior COVID-19 infection could improve patient protection, depending on the variant. As Omicron variants can escape prophylactic tixagevimab and cilgavimab, there is an urgent need to offer KTRs another mAb.

## Data availability statement

The datasets presented in this study can be found in online repositories. The names of the repository/repositories and accession number(s) can be found in the article/supplementary material.

## Ethics statement

The studies involving human participants were reviewed and approved by Data Protection Commission of the Assistance Publique-Hôpitaux de Marseille: reference PADS22-136, and Ethics Committee of the IHU Méditerranée Infection: number 2022–003. Written informed consent for participation was not required for this study in accordance with the national legislation and the institutional requirements.

## Author contributions

VM and BL designed the study. MV, CB, NO, AB, and SE carried out experiments. TR performed the statistical analysis. VM collected clinical data. PC analyzed the weekly incidence of major SARS-CoV-2 mutant and variant based on the genotyping. MV, CB, VM, and PC prepared the figures. VM wrote the first draft of the manuscript. All authors contributed to manuscript revision, read, and approved the submitted version.

## Funding

This work was supported by the French Government under the “Investments for the Future” program managed by the National Agency for Research (ANR), Méditerranée-Infection 10-IAHU-03.

## Conflict of interest

The authors declare that the research was conducted in the absence of any commercial or financial relationships that could be construed as a potential conflict of interest.

## Publisher’s note

All claims expressed in this article are solely those of the authors and do not necessarily represent those of their affiliated organizations, or those of the publisher, the editors and the reviewers. Any product that may be evaluated in this article, or claim that may be made by its manufacturer, is not guaranteed or endorsed by the publisher.
